# A Novel Cooling Device for Targeted Brain Temperature Control and Therapeutic Hypothermia: Feasibility Study in an Animal Model

**DOI:** 10.1007/s12028-016-0257-7

**Published:** 2016-02-29

**Authors:** E. Giuliani, S. Magnoni, M. Fei, A. Addis, R. Zanasi, N. Stocchetti, A. Barbieri

**Affiliations:** 1 Neuron Guard S.r.l., Via L. Castelvetro 15, 41124 Modena, Italy; 2 Department of Anesthesiology and Intensive Care, Ospedale Fondazione IRCCS, Ca’ Granda, Milan, Italy; 3 CRABCC, Biotechnology Research Center for Cardiothoracic Applications, Rivolta d’Adda, CR Italy; 4 Department of Engineering, University of Modena and Reggio Emilia, Modena, Italy; 5 Milan University, Milan, Italy; 6 Department of Anesthesiology and Intensive Care, University of Modena and Reggio Emilia, Modena, Italy

**Keywords:** Hypothermia, Brain temperature, Traumatic brain injury, Cardiac arrest, Stroke, Cooling collar

## Abstract

**Background:**

Therapeutic hypothermia (i.e., temperature management) is an effective option for improving survival and neurological outcome after cardiac arrest and is potentially useful for the care of the critically ill neurological patient. We analyzed the feasibility of a device to control the temperature of the brain by controlling the temperature of the blood flowing through the neck.

**Methods:**

A lumped parameter dynamic model, with one-dimensional heat transfer, was used to predict cooling effects and to test experimental hypotheses. The cooling system consisted of a flexible collar and was tested on 4 adult sheep, in which brain and body temperatures were invasively monitored for the duration of the experiment.

**Results:**

Model-based simulations predicted a lowering of the temperature of the brain and the body following the onset of cooling, with a rate of 0.4 °C/h for the brain and 0.2 °C/h for the body. The experimental findings showed comparable cooling rates in the two body compartments, with temperature reductions of 0.6 (0.2) °C/h for the brain and 0.6 (0.2) °C/h for the body. For a 70 kg adult human subject, we predict a temperature reduction of 0.64 °C/h for the brain and 0.43 °C/h for the body.

**Conclusions:**

This work demonstrates the feasibility of using a non-invasive method to induce brain hypothermia using a portable collar. This device demonstrated an optimal safety profile and represents a potentially useful method for the administration of mild hypothermia and temperature control (i.e., treatment of hyperpyrexia) in cardiac arrest and critically ill neurologic patients.

**Electronic supplementary material:**

The online version of this article (doi:10.1007/s12028-016-0257-7) contains supplementary material, which is available to authorized users.

## Introduction

Therapeutic hypothermia is recommended to improve the survival and neurological outcome of patients following cardiac arrest [1, 2]. However, there is a lack of consensus on the optimal target temperature, the timing of initiation, the duration of hypothermia, and the cooling methods [3]. Recent evidence from a randomized clinical trial suggests no additional benefits, in terms of survival and neurological outcome, cooling to 36 versus 33 °C, which indicates that mild hypothermia and avoiding hyperthermia could be sufficient to confer neuroprotection in out-of-hospital cardiac arrest patients [4]. In this and the other clinical trials designed to test the effects of hypothermia following cardiac arrest [4–7], hypothermia was generally obtained with aggressive cooling by means of methods such as intravascular and surface cooling devices. While whole-body cooling is an efficient method to reduce the brain temperature, it is however poorly tolerated without heavy sedation and pharmacological treatment of shivering. Whole-body cooling is also associated with several side effects, including coagulation disturbances, immunosuppression, and cardiovascular instability [8]. As hypothermia-induced neuroprotection derives from brain cooling and does not require to cool down the whole body, local brain cooling represents an attractive, viable and cost-effective alternative to whole-body hypothermia. Moreover, selective brain cooling is potentially applicable for prolonged clinical use, for brain selective mild hypothermia following cardiac arrest and prevent fever in critically ill neurologic patients. There is therefore growing interest in developing novel methods to effectively administer brain hypothermia and temperature control [8–10]. Accordingly, our objective was to analyze the feasibility of a portable cooling method to control the temperature of the brain via controlling the temperature of the blood flowing through the neck.

## Materials and Methods

Our study design incorporated three integrated phases: (1) the development of a thermodynamic model to simulate whole-body circulation system for a large animal; (2) testing of experimental hypotheses on the function of the neck as a heat exchanger for brain cooling in a large animal model, using our custom-designed portable cooling system; (3) and computational adaptation of the thermodynamic model to predict experimental outcomes for the human system.

### The Thermodynamic Model

**Fig. 1:**
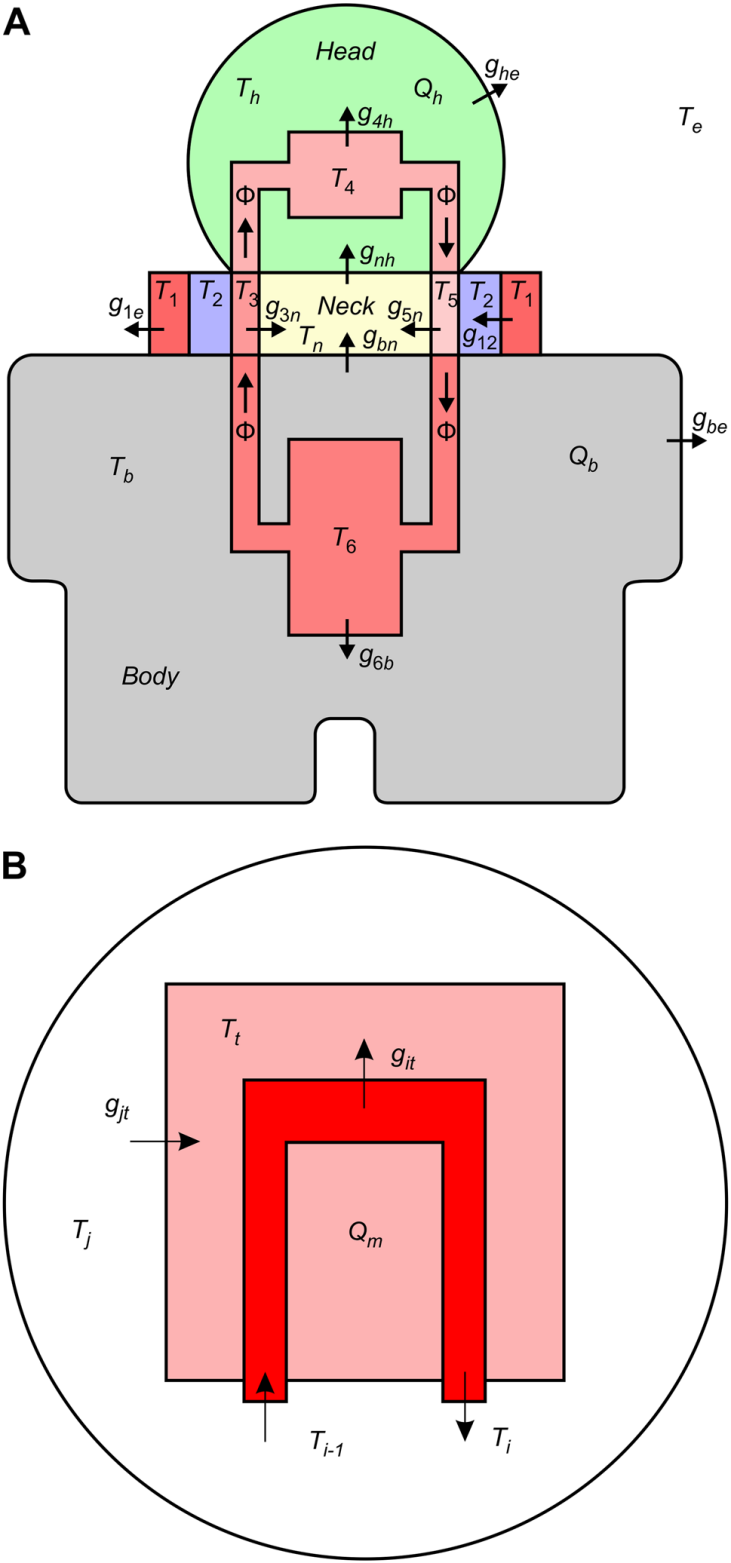
Schematic representation of the thermodynamic model of the whole human body (**a**), with the *inset* showing the model for a specific tissue (**b**). In this model, the head (h), neck (n), and body (b) are represented as separate compartments connected to each other by the cardiovascular system (in *red*). The temperature of each element (i.e., tissue or vessel) was considered to vary with time but to remain uniform within the tissue or vessel at any instant. *T*_1_ and *T*_2_ are the temperatures of the cooling elements of the collar, *T*_3_ and *T*_5_ are the temperatures of the blood flowing through the neck (in and out), while *T*_4_ and *T*_6_ refer to the temperatures of the blood in head and body, respectively. Other abbreviations: *T*_b_, *T*_h_, *T*_n_, *T*_e_—temperatures of the body, head, neck, and environment, respectively; *Q*_b_, *Q*_h_, *Q*_m_—metabolic heat of the body, head, and tissue, respectively; *g*_1e_, *g*_be_, *g*_he_, *g*_6b_, *g*_bn_, *g*_nh_, *g*_3n_, *g*_5n_, *g*_4h_, *g*_12_, *g*_jt_, *g*_it_—thermal conductivity between: the device and the environment, the body and the environment, the head and the environment, the blood and the body, the body and the neck, the neck and the head, the neck and the blood flowing to the head, the neck and the blood flowing back from the head, the blood and the head, the cooling elements of the collar, the environment and the tissue, the blood and the tissue, respectively; ϕ—blood flow within each specific segment of the cardiovascular system. In **b**, $T_{t }$ defines the tissue temperature, $T_{j }$ the temperature of an adjacent body (i.e., tissue, cooling element, or environment), and $T_{i - 1}$, $T_{i }$ the temperature of the blood entering and exiting the tissue. Note that the thermal conductivities $g_{tj} = \lambda_{t}  \frac{{S_{t} }}{{L_{t} }}$ and $g_{it} = h_{b} S_{v}$ are related to the conduction and the convection phenomena, respectively. While $\dot{Q}_{m}$ is the basal metabolic heat

A lumped parameter dynamic model, with one-dimensional heat transfer, was used to predict cooling effects and to test experimental hypotheses. In this model, the temperature of each element (i.e., tissue or vessel) was considered to vary with time but to remain uniform within the tissue or vessel at any instant. Within this whole-body thermodynamic model, we considered the head, neck, and body as separate compartments connected to each other by the cardiovascular system (Fig. 1a) [11]. Heat transfer in any one of these three compartments was described by the following two equations [12].

1 $$ \rho_{b} V_{i} c_{b} \frac{{{\text{d}}T_{i} }}{{{\text{d}}t}} = h_{b} S_{v} \left( {T_{t} - T_{i} } \right) + \rho_{b} c_{b} \phi_{b} \left( {T_{i - 1} - T_{i} } \right) $$

2 $$ m_{t} c_{t} \frac{{{\text{d}}T_{t} }}{{{\text{d}}t}} = \lambda_{t} \frac{{S_{t} }}{{L_{t} }}\left( {T_{j} - T_{t} } \right) + \dot{Q}_{m} , $$

The first thermodynamic equation describes the convective transmission of heat between blood and tissues, while the second equation describes the transmission of metabolic heat between tissues and the adjacent bodies (i.e., tissue, cooling element, or environment). As described in Eq. 1 (Fig. 1b), blood flows ($\rho_{b} V_{i} c_{b} \frac{{{\text{d}}T_{i} }}{{{\text{d}}t}}$) into a tissue with a convection mass $\dot{m}_{b} = \rho_{b} \phi_{b}$ (where $\rho_{b}$ is the blood density, $V_{i}$ is the volume, $c_{b}$ the specific heat and $\phi_{b}$ is the flow) and exchanges heat with that tissue through the walls of the vessels, depending on the conductivity of the tissue ($g_{it} = h_{b} S_{v}$). The blood capability to absorb heat from the tissue is described by its specific heat $c_{b}$ and convective coefficient $h_{b}$. The blood enters at a temperature of $T_{i - 1}$, removes heat from the tissue at temperature $T_{t}$ and exits at temperature $T_{i}$ [13].

The tissue exchange of heat with adjacent bodies (i.e., tissue, cooling element, environment) depends on $T_{j} - T_{t}$, where $T_{j }$ is the temperature of an adjacent body (i.e., tissue, cooling element, or environment), $T_{t }$ defines the tissue temperature, and its thermal conductivity $\lambda_{t}$ (that is the property of the tissue to conduct heat) and the heat produced by the metabolism as indicated in Eq. 2 (Fig. 1b). The tissue exchanges metabolic heat ($\dot{Q}_{m}$) through conduction ($g_{tj} = \lambda_{t}  \frac{{S_{t} }}{{L_{t} }}$), with adjacent bodies (i.e., tissue, cooling element, environment).

For metabolic heat generation the basal metabolic heat $\dot{Q}_{m}$ is used in the model [14].

The parameters used in the simulation of our model are listed in (Supplement Table).

### The Cooling System

**Fig. 2:**
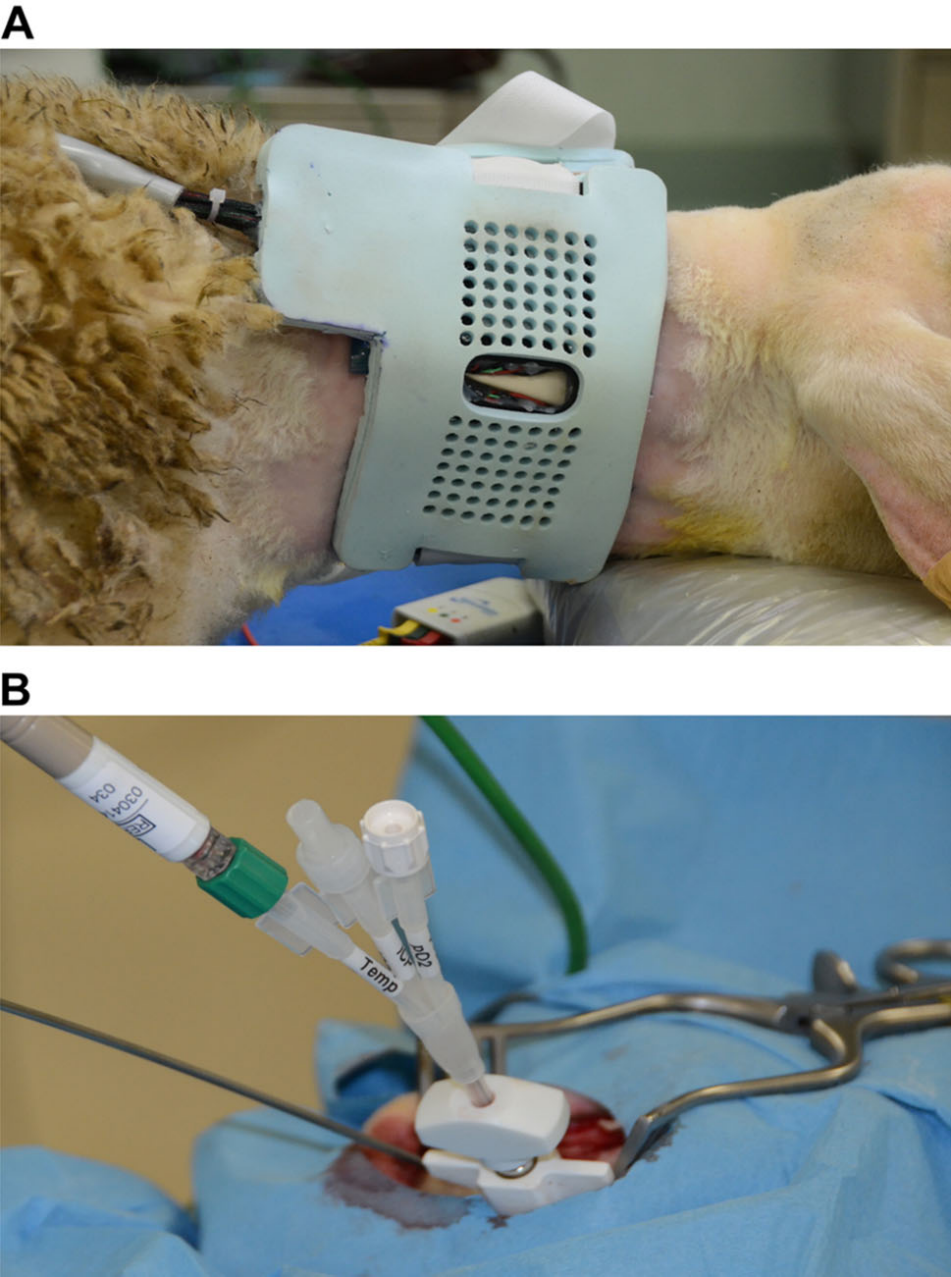
**a** Cooling collar wrapped around the neck of sheep after the induction of anesthesia. The neck of the animal was shaved and covered with a thin layer of conductive gel to improve heat transfer from the neck to the device. Individual cooling elements were placed on the tissues overlying carotid arteries (identified by Doppler scanning). The device was connected to a control unit that controlled each cooling element on the basis of the temperature feedback. **b** brain temperature monitoring system consisted of an in situ PT-100 ATEX sensor. The sensor was inserted 1 cm into the white matter between the parietal and occipital lobe with a sagittal postero-anterior orientation through a burr drilled into the right occipital bone, using a 1.5-mm ball tool (Medtronic Midas Rex surgical drill). The burr hole was sealed with sterile bone wax. In the foreground a LiCox^®^ temperature sensor used to assess the consistency of the temperature recordings of the reference sensor

A surface cooling system was designed to target the carotid arteries of the neck. The cooling system consisted of a flexible collar, powered by a 60 W source. Considering an average efficiency of 60 % for the system, calculated from the manufacturer’s datasheets for the cooling elements, a local heat removal of 36 W was estimated. Figure 2a illustrates the cooling collar wrapped around the sheep neck.

### Experimental Study on a Large Animal Model

All applicable institutional and/or national guidelines for the care and use of animals were followed. Housing, husbandry and experimental procedures were approved and carried out in conformity with the guidelines of the Review Board for animal studies at the Istituto Superiore di Sanità (ISS), and the national guidelines (D.Lgs 26/2014) of the Italian Ministry of Health and International Laws and Policies (EEC Council Directive, 2010/63/UE). The study was authorized by the institutional and ethics review board of the Italian Ministry of Health (trial registration 351/2015-PR 05/11/2015). The experiments were carried out at CRABCC, Biotechnology Research Center for Cardio-Thoracic Applications (Rivolta D’Adda, Italy).

The prototype of the cooling system was tested on 4 adult Bergamasca sheep (*Ovis aries*, Bergamasca breed, 1-year-old, 65–70 kg body weight and all female). After sedation, general anesthesia was induced by ketamine (2–5 mg/kg intravenous IV); combined with midazolam (0.5 mg/kg IV) administered by injection into the external jugular vein. As soon as practical, each animal was intubated and mechanically ventilated. Anesthesia was maintained with 2–3 % isoflurane in 100 % oxygen. An orogastric tube was positioned to evacuate gaseous and liquid gastric contents. Blood pressure was monitored invasively during the procedure through the cannulation of the auricular artery in two animals. A thermal blanket was wrapped around the body of the animal to control the body temperature during the procedure. The head was then stabilized with adhesive patches, and a burr hole drilled into the right occipital bone, using a 1.5-mm ball tool (Medtronic Midas Rex surgical drill), for the placement of the reference brain temperature sensor (PT-100 ATEX sensor) 1 cm into the white matter between the parietal and occipital lobe with a sagittal postero-anterior orientation. The brain sensor in place is shown in Fig. 2b. The parenchymal position of the catheter into the cerebral parenchyma was confirmed by post-mortem inspection of the brain and no visible signs of brain hemorrhages were noted in any part of the brain. The burr hole was sealed with sterile bone wax and the head was covered with surgical blankets to reduce thermal dispersion. Body temperature was measured using a rectal/esophageal probe. ECG, pulse oximetry, capnography, invasive blood pressure, and brain and esophageal temperatures were monitored by an anesthesiologist throughout the procedure and digitally recorded.

The neck was completely shaved so each carotid artery was identified using echo Doppler scanning for the placement of the two refrigerating elements. The device was activated and programmed to reduce skin temperature no lower than 5 °C. Local, brain, and body temperatures were recorded continuously (1 Hz sampling rate, duration of 3600 s). The system was controlled through feedback using local temperature sensors. Brain (in situ PT-100 sensor) and body (esophageal probe) temperatures were recorded continuously (1 Hz sampling rate, duration of monitoring 3600 s). At the end of the experiment, the cooling collar was removed and the skin under the cooling elements visually examined. The animals, still under anesthesia, were euthanized by IV injection of pentothal sodium (50 mg/kg) and potassium chloride (20 mg/kg).

### Statistical Analysis

Normally distributed variables are expressed as mean ± standard deviation (SD). The Shapiro–Wilk *W* test was used to assess the normality of the distribution of temperature recordings. Between-group comparisons were evaluated using two-tailed, Student’s *t* test, with a *P* value <0.05 considered to be significant. All statistical analyses were performed using Data and Statistical Software (STATA© software version 10.0, Stata Corp., TX, USA).

## Results

**Fig. 3:**
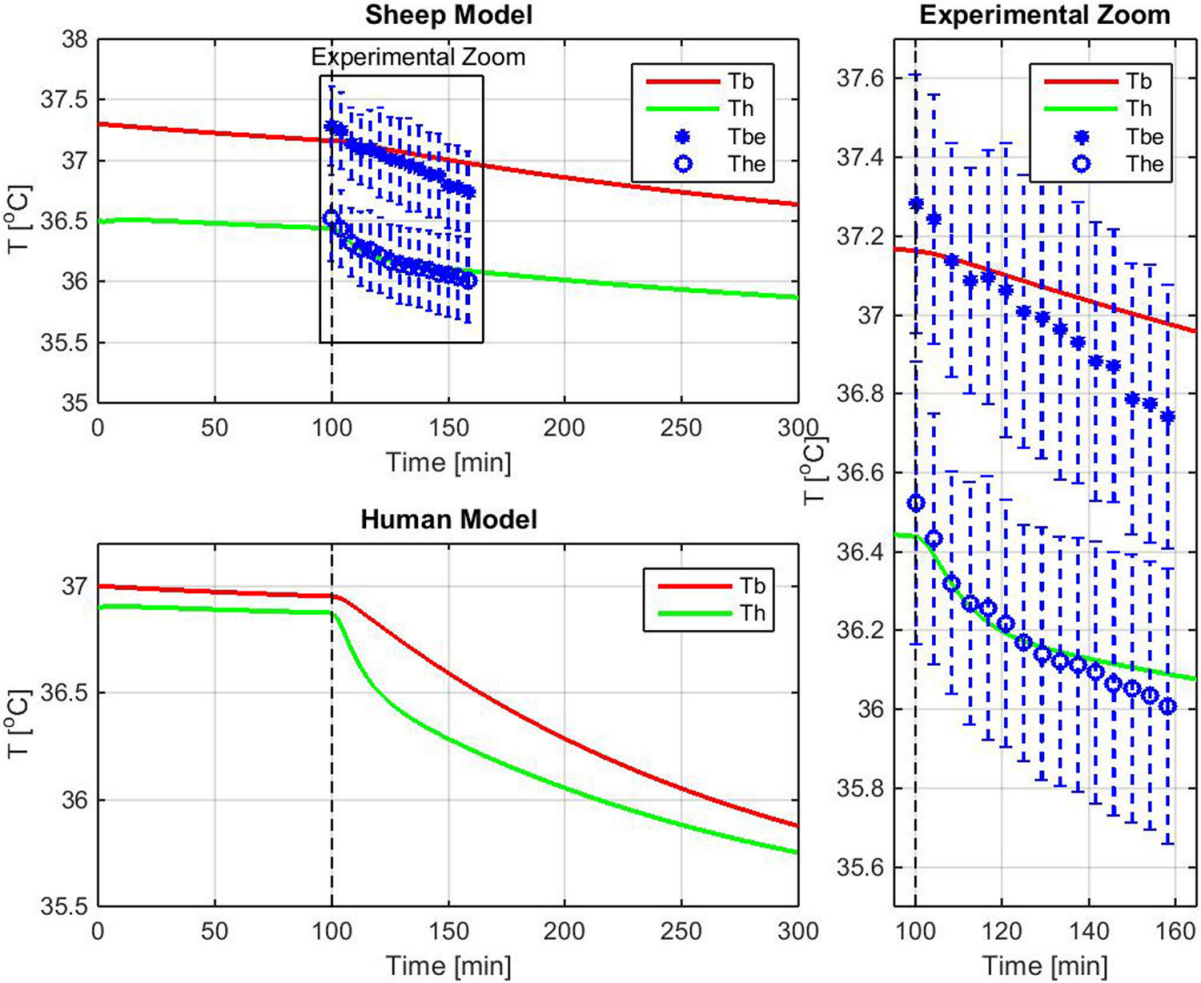
Sheep and human thermodynamic models, showing simulated values of the time-dependent cooling of the brain (Th) and body (Tb) induced by neck cooling. For the sheep, the simulated data were fitted to the experimental data with their confidence intervals (Tb_e_ and Th_e_, *blue symbols*). The Experimental Zoom panel shows the experimental temperature recordings and simulated temperatures with a better resolution. The time from 0 to 100 min is the setting time required by the model to reach the steady state. The sheep model confirmed lower temperatures of the brain compared to the body, with baseline values (at 100 min) of 36.5 (0.4) °C for the brain and 37.3 (0.3) °C for the body. The experimental cooling rate for the brain was 0.6 (0.2) °C/h in line with the simulation (*green line*, in the zoom panel), while the temperature reduction of the body was 0.6 (0.2) °C/h, slightly higher than predicted (*red line*, in the zoom panel). The temperature variation, before and after application of the cooling collar (i.e., at baseline and after 60 min), was statistically significant, both for the brain (*P* = 0.0072) and the body (*P* = 0.0090). Temperatures are expressed in °C and time in min

Model-based simulation of the time series of brain and body temperatures values is reported in Fig. 3, for both the sheep and human thermodynamic model. Note that the experimental data and their confidence intervals are compared with the simulation in the Experimental Zoom box of Fig. 3. The sheep model predicted lower baseline temperature of the brain than the body, while for the human, comparable baseline temperatures were predicted for the brain and body. Both models predicted a lowering of the temperature of the brain and body following onset of cooling from steady state conditions (i.e., after a settling time of 100 min the cooling system started to work). The thermodynamic model for the sheep predicted a cooling rate of 0.4 °C/h for the brain and 0.2 °C/h for the body due to the back flow of colder blood into the circulatory system.

To test the validity of the predicted data, the time series of brain and body cooling temperatures were evaluated in 4 animals (Table 1). Baseline temperatures confirmed a lower temperature of the brain compared to the body, with a mean baseline temperature of 36.5 (0.4) °C for the brain and 37.3 (0.3) °C for the body. The experimental cooling rate for the brain was 0.6 (0.2) °C/h in line with the simulation, while the temperature reduction of the body was slightly higher than predicted, 0.6 (0.2) °C/h, likely due to the metabolic reduction induced by general anesthesia, not included into the simulation (Fig. 3). Indeed, the model used the basal metabolic heat $\dot{Q}_{m}$ of 1200 kcal. Nevertheless, simulated temperatures fell within the confidence interval of experimental data. As illustrated in Table 1, the temperature variation, before and after application of the cooling collar (i.e., at baseline and after 60 min), was statistically significant, both for the brain (*P* = 0.0072) and the body (*P* = 0.0090).

**Table 1:** Average brain and body temperatures at baseline and after 3600 s of neck cooling

	Baseline temperature (°C)	Temperature at 60 min (°C)	*P* value
Brain			
Sheep 1	36.0	35.6	
Sheep 2	36.5	35.9	
Sheep 3	36.8	36.1	
Sheep 4	36.8	36.4	0.0060
Body			
Sheep 1	36.9	36.3	
Sheep 2	37.1	36.6	
Sheep 3	37.6	36.8	
Sheep 4	37.5	37.1	0.0067

Temperatures were recorded with 1 Hz sampling rate; values are the moving average at baseline and 3600 s

Heart rate (HR) and peripheral saturation of oxygen (SpO_2_) were continuously monitored in all animals; invasive arterial blood pressure (IABP) recordings were available for two animals. Average baseline HR, IABP, and SpO_2_ were 102.0 ± 13.6 bpm, 98.5 ± 9.2 mmHg, 98.0 ± 2.3 % and after 60 min of cooling 104.7 ± 6.2 bpm, 92.5 ± 10.6 mmHg, 97.8 ± 1.5 %: the differences were not statistically significant for HR and SpO_2_ (*P* value 0.7252, 0.8619 respectively); statistical analysis was not performed for IABP due to the limited number of available recordings.

The skin temperature under the cooling elements was 14.5 (0.5) °C. There were no significant systemic side effects of cooling during the experiments and no local lesions at the interface between the cooling elements and the skin of the neck.

By fitting the experimental data to the thermodynamic model, we would predict a cooling rate for the brain of 0.64 and 0.43 °C/h for the body for a 70 kg adult human subject (Fig. 3).

## Discussion

The present work suggests that effective brain cooling can be achieved with an external cooling collar applied to the neck surface to cool down the arterial vessels tributaries of cerebral circulation, with negligible systemic side effects and no skin lesions. This device has a sophisticated temperature management system, able to regulate the intensity and duration of brain cooling by modulating the temperature difference between the individual cooling elements and the underlying tissues, blood vessels, and ultimately blood. According to the thermodynamic model, in humans, the efficiency of the neck as heat exchanger is expected to be similar or even higher compared to the sheep.

The portable device for targeted brain cooling we developed could be deployed in the field to induce early on-site hypothermia, used for in-hospital maintenance of mild hypothermia in the same patients, or for the treatment of fever and temperature control in critically ill neurologic patients.

Although early on-site hypothermia is an attractive approach to improve outcomes after cardiac arrest, the results of clinical studies on out-of-hospital hypothermia provided conflicting results, possibly because of suboptimal methods of inducing hypothermia [7, 9]. The adoption of a minimally invasive, portable cooling device such this could offer first responders the opportunity to administer effective hypothermia while providing emergency care, and provide a *therapeutic bridge* until further treatments can be accessed.

The present cooling system represents an attractive, viable and cost-effective alternative to surface and intravascular cooling methods for subjects suffering from cardiac arrest and acute brain injury. While the prevention of fever and the maintenance of normothermia are recommended in the acute phase following traumatic brain injury, stroke, and subarachnoid hemorrhage [15–17], the optimal method to do so has not been identified yet. Hypothermia is also useful for the control of elevated intracranial pressure in patients with traumatic brain injury, but this maneuver may be associated with detrimental side effects and result in worse outcome [18]. Hence, another potential application of the present device is as a “more gentle” system to control the brain temperature and help in the treatment of elevated intracranial pressure.

The thermodynamic model indicates that a target differential temperature of 1–2 °C can be attained within 2–4 h with this cooling device. Compared to whole-body cooling methods, this is less effective in reducing the body temperature, however with an improved safety profile. It is well established that intravascular devices may be associated to complications such as vascular thrombosis [19, 20], whereas the infusion of large volumes of cold fluids may induce volume overload [7]. The experimental results suggest a good safety profile of the device also in terms of local skin injury. Sheep’s skin, however, is protected by a layer of lanolin, which makes it more resistant than human skin to environmental hazards like low temperature. Therefore, the effects of the device on local skin will need to be carefully tested in humans, to determine the minimum feasible temperature in a clinical scenario.

Furthermore, safety monitoring will be fundamental when moving on human subjects, to carefully check for potential detrimental effects of the cooling collar on the arterial blood flow dynamics (i.e., the transition from laminar to turbulent flow), which may result in blood hypercoagulability. Adequate brain imaging techniques and/or non-invasive monitoring of the cerebral blood flow for the early detection of ischemia (e.g., with echo color Doppler or Near Infrared Spectroscopy NIRS) are therefore recommended when conducting human studies.

Shivering is the most common effect of hypothermia, as thermoceptors are mostly located on the exterior layers of the human body, especially in the dermis. Surface cooling devices suffer from a more intense thermoceptor stimulation than invasive cooling systems. The limited surface of our cooling system has the advantage of reducing the number of thermoceptors involved with a potentially positive effect on shivering.

Notably, the rate of temperature reduction of 0.6 °C/h obtained with the cooling collar is better than 0.25 °C/h reported for other cooling methods utilized in a large trial of 900 patients [4].

A number of devices have been investigated for elective brain cooling, with the aim to minimize the side effects of whole-body cooling. At present time, the evidence on the clinical applicability of these methods (e.g., cooling helmets, nasopharyngeal cooling and carotid intra-arterial cooling) is limited, due to safety issues or very little efficiency [10, 21].

The current design of our cooling system did not produce a selective effect on the brain, as it was however predicted by the model, possibly due to the constant backflow of cooled blood from the brain to the body, or to the effects of general anesthesia on the basal metabolism of both brain and body. However, this effect can be easily counterbalanced by measures to limit body cooling, such as the use of thermal blankets. Therefore, the cooling collar has the potential to create and maintain separate temperature zones for the head and the body and, thereby, limit the negative effects of body hypothermia while optimizing the therapeutic effect of hypothermia and temperature control on the target organ.

In humans, the model still predicts a more selective effect on the brain compared to the body, with a temperature difference of 0.2 °C/h. In humans, unlike sheep, both carotid and vertebral arteries can be targeted for cooling, which, in combination with higher cerebral blood flow, are expected to increase the efficiency of the neck as heat exchanger. In addition, a more selective effect might be related to the impact of general anesthesia on basal metabolism [22]. The hypothesis is that under anesthesia sheep’s body, whose mass is relatively bigger compared to the brain, may have cooled down more, thus counteracting the selective effects of the cooling device, whereas in humans this effect is likely diminished. Second, cooling during the first hours of general anesthesia is partly due to redistribution from core to peripheral tissues; body peripheral tissue mass is considerably larger than head and may have absorbed proportionally more heat [22]. In humans, however, where the mass of the brain is larger compared to the body, we expect an opposite effect. Most importantly, these data will have to be confirmed in a human study.

The device did not produce any significant effect on heart rate and blood pressure, compared to similar surface devices [23]. These data, if confirmed also in humans, would attest a more favorable safety profile compared to other surface cooling devices [10].

The device was programmed to reduce skin temperature no lower than 5 °C, a safety threshold to prevent local freezing. In the experimental setting, however, local skin temperatures never reached this limit, being on average about 14.5 °C. These findings suggest that the device, still a prototype, may need more cooling power to reach the lowest programmed skin temperature, since the carotid arteries act as a continuous source of heat that counterbalances the cooling effect of the collar. This is also the main functional mechanism of the collar—removing heat from the blood supply of the brain while it flows through the neck. Accordingly, when the collar was positioned not directly in contact with the carotid arteries (i.e., in a portion of the neck far from the vessels) the lowest temperature was reached within about 10 min from activation. These data, if confirmed in humans, will be important in the definition of the temperature feedback control algorithm. Engineering work is under way to refine the prototype and set up a final, more potent device.

Due to the unavailability of suitable animal models to test the effectiveness of the present neck cooling collar, we used a sheep model despite its limitations which include: brain size that is approximately 1/10th of the size of a human brain; specific anatomical features of the neck that allows only the carotid arteries to be reached by the direct cooling action [24]; and a differential in body-to-brain temperatures, with body temperature frequently higher than that of the brain [25]. Based on anatomical and physiological differences between sheep and humans, the effectiveness of the cooling collar would arguably be higher in humans than in sheep.

Interestingly, the thermodynamic model predicted comparable temperatures for the brain and body in humans, in contrast to the higher brain-to-body temperatures reported in patients with acute brain damage [26]. It is well documented that the regulation of brain temperature may be impaired after major brain injury, whereas little is known about healthy conditions [27]. Additional investigation will be needed into the conditions of human brain injury, as there are no models of brain injury in sheep, whose data can be effectively used for human research.

Our study has several limitations including the limited number of animals and the aforementioned limits of the model itself. Regrettably, to date, we are not aware of a suitable alternative whose characteristics, in terms of body mass, anatomy of the neck and size of the brain, are comparable to those of humans.

In the current experimental setting, the prototype devices were tested for a continuous use of 60 min due to the cooling power available and the characteristics of the heat dissipation system. Pilot data were recorded indicating that the system did not show signs of performance degradation even for longer periods (120 min).

In conclusion, our experiments demonstrate the feasibility of using a non-invasive method to induce brain hypothermia using the neck as a heat exchanger. However, there is still no enough evidence that a brain selective effect can be achieved. The portable cooling collar that we developed could offer an advantage in terms of portability and early operability also in out-of-hospital environments. The next step is to undertake a trial of feasibility in humans.

Proof of superiority of any cooling method above others is still lacking, and there are currently no formal cost-benefit analyses.

## Electronic supplementary material

Below is the link to the electronic supplementary material.

Supplementary material 1 (DOCX 16 kb)
