# Supplementary material for: A Novel Cooling Device for Targeted Brain Temperature Control and Therapeutic Hypothermia: Feasibility Study in an Animal Model
Source: Neurocrit Care. 2016 Feb 29;25(3):464–72. doi: 10.1007/s12028-016-0257-7 (PMC5138276; doi:10.1007/s12028-016-0257-7)
Supplement: Supplementary file 1 — Supplementary material 1 (DOCX 16 kb) [file 12028_2016_257_MOESM1_ESM.docx]

SUPPLEMENT TABLE

Tissues characteristics, in terms of resistance and heat capacity (ref. IT’IS Foundation)

| Parameter | Symbol | Sheep | Human |
| --- | --- | --- | --- |
| Blood density | $\rho_{b}$ | $1049 \left[ \frac{kg}{m^{3}} \right]$ | $1600 \left[ \frac{kg}{m^{3}} \right]$ |
| Blood specific heat | $c_{b}$ | $3680 \left[ \frac{J}{kg K} \right]$ | $3850 \left[ \frac{J}{kg K} \right]$ |
| Blood thermal conductivity | $\lambda_{b}$ | $0.57 \left[ \frac{W}{m K} \right]$ | $0.57 \left[ \frac{W}{m K} \right]$ |
| Blood viscosity | $\mu_{b}$ | $3\cdot{10}^{-3} \left[ \frac{kg}{m s} \right]$ | $3\cdot{10}^{-3} \left[ \frac{kg}{m s} \right]$ |
| Brain blood flow | $\phi_{b}$ | $3.33\cdot{10}^{-6} \left[ \frac{m^{3}}{s} \right]$ | $1.09\cdot{10}^{-5} \left[ \frac{m^{3}}{s} \right]$ |
| Number of arteries | $n_{a}$ | 2 | 4 |
| Number of veins | $n_{v}$ | 2 | 2 |
| Arteries and veins length in contact with the cooling elements | ${l_{a}, l}_{v}$ | $4\cdot{10}^{-2} \left[ m \right]$ | $4\cdot{10}^{-2} \left[ m \right]$ |
| Arteries diameter | $d_{a}$ | $4\cdot{10}^{-3} \left[ m \right]$ | $5\cdot{10}^{-3} \left[ m \right]$ |
| Veins diameter | $d_{v}$ | $13.3\cdot{10}^{-3} \left[ m \right]$ | $12\cdot{10}^{-3} \left[ m \right]$ |
| Brain mass | $m_{H}$ | $0.5 \left[ kg \right]$ | $1.5 \left[ kg \right]$ |
| Blood volume in the brain | $V_{H}$ | $8\cdot{10}^{-5} [m^{3}]$ | $1.5\cdot{10}^{-4} [m^{3}]$ |
| Brain specific heat | $c_{H}$ | $3700 \left[ \frac{J}{kg K} \right]$ | $3700 \left[ \frac{J}{kg K} \right]$ |
| Body mass | $m_{B}$ | $55 \left[ kg \right]$ | $70 \left[ kg \right]$ |
| Blood volume in the body | $V_{B}$ | $3.2\cdot{10}^{-3} [m^{3}]$ | $4.1\cdot{10}^{-3} [m^{3}]$ |
| Body specific heat | $c_{B}$ | $3138 \left[ \frac{J}{kg K} \right]$ | $3138 \left[ \frac{J}{kg K} \right]$ |
| Neck mass | $m_{B}$ | $2 \left[ kg \right]$ | $1 \left[ kg \right]$ |
| Neck specific heat | $c_{H}$ | $3680 \left[ \frac{J}{kg K} \right]$ | $3680 \left[ \frac{J}{kg K} \right]$ |
| Skin thermal conductivity | $\lambda_{s}$ | $0.47 \left[ \frac{W}{m K} \right]$ | $0.47 \left[ \frac{W}{m K} \right]$ |
| Fat thermal conductivity | $\lambda_{f}$ | $0.21 \left[ \frac{W}{m K} \right]$ | $0.21 \left[ \frac{W}{m K} \right]$ |
| Muscle thermal conductivity | $\lambda_{m}$ | $0.51 \left[ \frac{W}{m K} \right]$ | $0.51 \left[ \frac{W}{m K} \right]$ |
| Bone thermal conductivity | $\lambda_{o}$ | $0.75 \left[ \frac{W}{m K} \right]$ | $0.75 \left[ \frac{W}{m K} \right]$ |
| Wool thermal conductivity | $\lambda_{w}$ | $0.033 \left[ \frac{W}{m K} \right]$ | $0.033 \left[ \frac{W}{m K} \right]$ |
| Wool thickness | $l_{B}$ | $2\cdot{10}^{-3} \left[ m \right]$ | $1\cdot{10}^{-7} \left[ m \right]$ |
| Metabolic rate | $\dot{Q_{m}}$ | $1200 \left[ kcal \right]$ | $2000 \left[ kcal \right]$ |
